# Supplementary material for: Effects on Metabolism in Astrocytes Caused by cGAMP, Which Imitates the Initial Stage of Brain Metastasis
Source: Int J Mol Sci. 2021 Aug 21;22(16):9028. doi: 10.3390/ijms22169028 (PMC8396466; doi:10.3390/ijms22169028)
Supplement: Supplementary file 1 [file ijms-22-09028-s001.zip › Supplement Fig S1_S2_0805.pdf]

Supplement Fig. S1

Amount of cGAMP detected in astrocytes after treatment with various lipid nanoparticles (LNPs) and cGAMP complexes

Sample			cGAMP [ng/1x10^6 cells]
DOPC50	6hr	cGAMP2	1.81
		cGAMP8	N.D.
	18hr	cGAMP2	4.30
		cGAMP8	5.79
DOPC200	6hr	cGAMP2	N.D.
		cGAMP8	N.D.
	18hr	cGAMP2	N.D.
		cGAMP8	5.25
DOPE50	6hr	cGAMP2	7.62
		cGAMP8	12.34
	18hr	cGAMP2	7.21
		cGAMP8	13.52
DOPE200	6hr	cGAMP2	3.06
		cGAMP8	5.62
	18hr	cGAMP2	4.35
		cGAMP8	4.02
Lipofectamine	6hr	control	N.D.
		cGAMP2	N.D.
		cGAMP8	N.D.
	18hr	cGAMP2	N.D.
		cGAMP8	N.D.

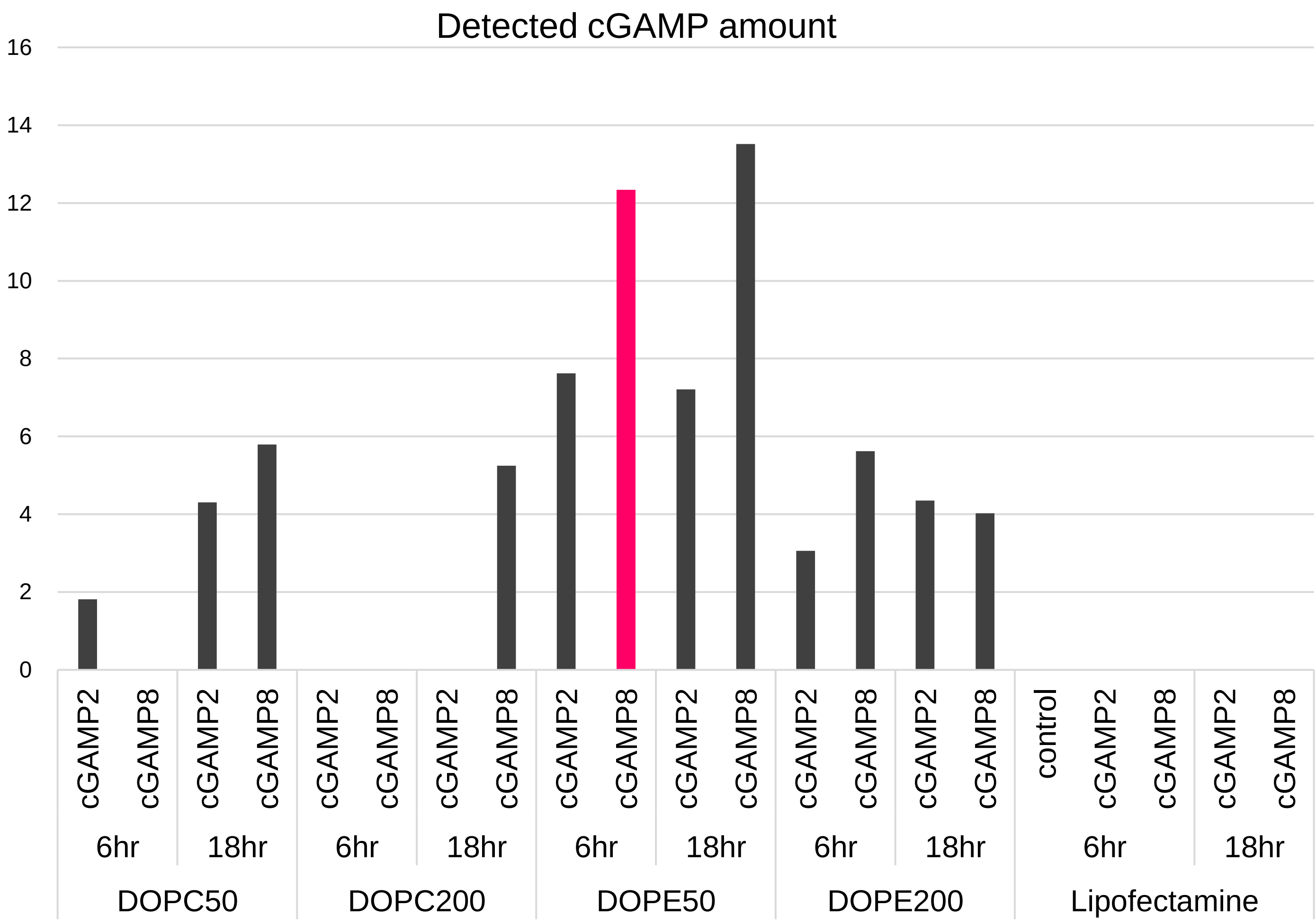

3 types of LNPs were prepared, DOPC, DOPE and Lipofectamine 3000. The ratio of lipid (nmol) to cGAMP (μg) was 50 and 200. cGAMP incorporation into LNPs was 2 or 8 μg/well, and the incorporation time into cells was 6 or 18 hours. Data are shown as mean values of duplicates. N.D.; not detected. The red bar condition (DOPE, 6hr, cGAMP 8 μg) was selected in the present study.

Supplement Fig. S2

Intracellular metabolites regarding glucose metabolism after $^{13}C6$-glucose incorporation normalized as z-score

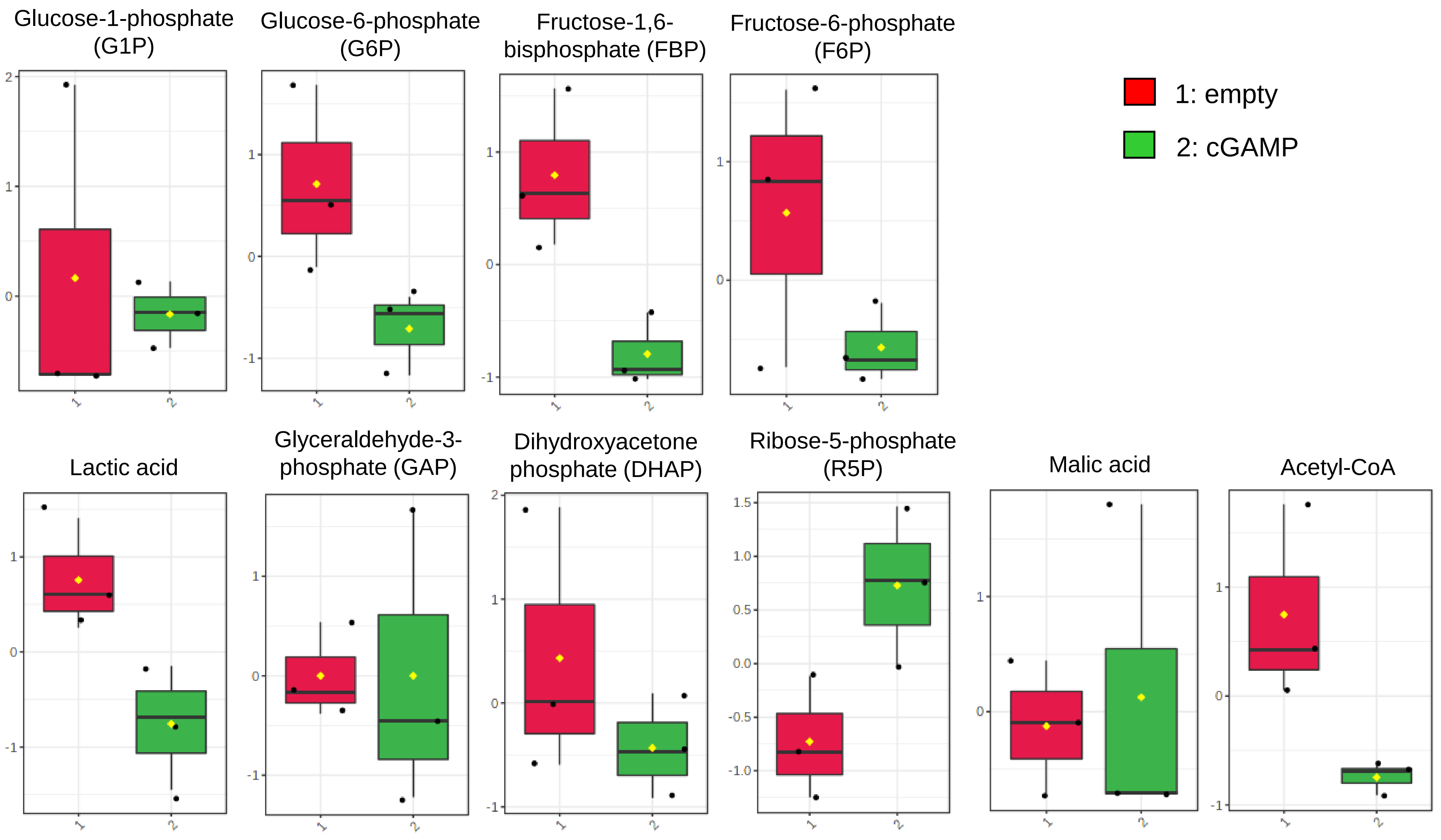

The most abundant isotopomer in each compounds are shown. G1P, G6P, FBP, and F6P represent $^{13}C6$ isotopomer. Lactic acid, GAP and DHAP represent $^{13}C3$ isotopomer, R5P represents $^{13}C5$ isotopomer, Malic acid, Acetyl-CoA represent $^{13}C2$ isotopomer.
