# Supplementary material for: Effects on Metabolism in Astrocytes Caused by cGAMP, Which Imitates the Initial Stage of Brain Metastasis
Source: Int J Mol Sci. 2021 Aug 21;22(16):9028. doi: 10.3390/ijms22169028 (PMC8396466; doi:10.3390/ijms22169028)
Supplement: Supplementary file 1 [file ijms-22-09028-s001.zip › Table S1_Nanoparticle characteristics.pdf]

Table S1 Nanoparticle characteristics of ssPalm-cGAMP or ssPalm-empty complex.

Lipids	Lupid / cGAMP [nmol / µg]	cGAMP [µg]	Size [d.nm]*	PdI*	Zeta Potential [mV]*
DOPC	50	0	101.6	0.351	-0.965
		2	92.9	0.327	-5.15
	200	0	64.19	0.397	-2.65
		2	107.5	0.444	-2.61
DOPE	50	0	110.8	0.413	-5.39
		2	97.94	0.302	-8.18
	200	0	109.8	0.536	-5.81
		2	83.06	0.469	-5.65

For searching optimal condition of ssPalm-cGAMP, 8 kinds of group were prepared: dioleoyl-sn-glycero-phosphatidylcholine (DOPC) or dioleoyl-sn-glycero-phosphoethanolamine (DOPE) was used as the lipid component. The cGAMP/lipid ratio was tried for 50 nmoL/µg and 200 nmoL/µg. cGAMP was included 2 µg and distilled water is substituted in each control group. *Size, PdI, and Zeta Potential were mesured by dynamic light scattering (Zetasizer Nano ZS, Malvern).
